# Bifunctional fluorescent probes for detection of amyloid aggregates and reactive oxygen species

**DOI:** 10.1098/rsos.171399

**Published:** 2018-02-07

**Authors:** Lisa-Maria Needham, Judith Weber, James W. B. Fyfe, Omaru M. Kabia, Dung T. Do, Ewa Klimont, Yu Zhang, Margarida Rodrigues, Christopher M. Dobson, Sonia Ghandi, Sarah E. Bohndiek, Thomas N. Snaddon, Steven F. Lee

**Affiliations:** 1 Department of Chemistry, University of Cambridge, Lensfield Road, Cambridge, CB2 1EW, UK; 2 Department of Physics, University of Cambridge, JJ Thomson Avenue, Cambridge, CB3 0HE, UK; 3 Cancer Research UK Cambridge Institute, University of Cambridge, Li Ka Shing Centre, Robinson Way, Cambridge, CB2 0RE, UK; 4 Department of Chemistry, Indiana University, 800 E. Kirkwood Avenue, Bloomington, IN 47405, USA; 5 Department of Molecular Neuroscience, Institute of Neurology, University College London, Queen Square, London WC1N 3BG, UK

**Keywords:** fluorophores, ROS, sensors, protein aggregation, single-molecule imaging, Thioflavin T

## Abstract

Protein aggregation into amyloid deposits and oxidative stress are key features of many neurodegenerative disorders including Parkinson's and Alzheimer's disease. We report here the creation of four highly sensitive bifunctional fluorescent probes, capable of H_2_O_2_ and/or amyloid aggregate detection. These bifunctional sensors use a benzothiazole core for amyloid localization and boronic ester oxidation to specifically detect H_2_O_2_. We characterized the optical properties of these probes using both bulk fluorescence measurements and single-aggregate fluorescence imaging, and quantify changes in their fluorescence properties upon addition of amyloid aggregates of α-synuclein and pathophysiological H_2_O_2_ concentrations. Our results indicate these new probes will be useful to detect and monitor neurodegenerative disease.

## 1. Introduction

**Figure 1.**
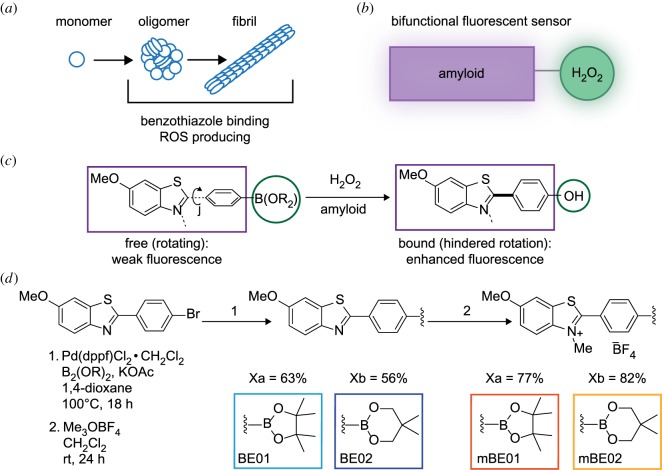
(*a*) Protein misfolding pathway of amyloid fibril formation. (*b*) Concept of the bifunctional fluorescence sensor design; one part detects amyloid, the second component undergoes chemical modification following reaction with H_2_O_2_. Both of these elements lead to a change in the optical properties of the sensor. (*c*) Scheme illustrating modifications of optical properties. (*d*) Synthetic scheme for the dyes- BE01, BE02, mBE01 and mBE02. Reaction conditions and reagents (1 and 2) are listed on the left.

Neurodegenerative diseases, such as Parkinson's and Alzheimer's disease, are characterized pathologically by the presence of amyloid deposits following protein misfolding, aggregation and neuronal loss in vulnerable brain regions [1]. Pathological aggregated proteins emerge by the misfolding of monomeric proteins into intermediate oligomeric structures, which polymerize into insoluble, highly organized structures, known as amyloid fibrils (figure 1*a*). There is a growing interest in the idea that these intermediate oligomeric species may play a crucial role in cellular toxicity in the disease [2]. Molecular alterations occur in the early stages of neurodegenerative disease and long precede symptomatic onset that accompanies neuronal death [3,4]. Thus, visualization of these early processes at the molecular level can be used to detect, predict and monitor the development of disease. In addition to protein aggregation, oxidative stress has been widely reported to occur in *in vitro* and *in vivo* models of neurodegenerative disease. Furthermore, oxidative damage is seen in post-mortem brain. Together, this evidence proposes a key role for oxidative stress in the pathogenesis of such diseases [5]. The interaction between α-synuclein (*α*Syn) and oxidative stress in neurodegeneration has been shown to be bidirectional, with oxidative stress promoting the aggregation of proteins such as *α*Syn and the misfolding of *α*Syn generates oligomers associated with increased reactive oxygen species (ROS) production [6,7]. Therefore, two crucial factors common in most neurological pathologies are (1) the accumulation of abnormally aggregated proteins and (2) high levels of ROS [8]. Abnormally aggregated proteins are commonly visualized and quantified using fluorescence-based methods. The main structural hallmark of amyloid-like aggregates is an ordered arrangement of β-sheets [9,10]; this structural feature facilitates specific binding of many fluorescent compounds including the widely used benzothiazole compound, thioflavin t (ThT) [11]. ThT undergoes an increase in fluorescence quantum yield (*Φ*_Fl_) of several orders of magnitude upon incorporation into these β-sheet containing species [12]. This phenomenon occurs due to physical suppression of a non-fluorescent twisted intramolecular charge transfer (TICT) state as a consequence of rapid rotation about the C–C bond between benzothiazole and *N*,*N*-dimethylaniline rings (figure 1*c*) [12]. This fluorescence ‘turn-on' makes ThT an essential probe of β-sheet in both the abundant fibrillar species and the rarer, potentially toxic oligomeric species [13,14].

ROS encompass a vast array of chemical compounds including singlet oxygen, hydroxyl radicals, peroxides and superoxides [15]; they are endogenously produced, reactive molecules and free radicals derived from molecular oxygen. In the brain they are principally generated by the electron transport chain in the mitochondria, the enzymes NADPH oxidase, xanthine oxidase and monoamine oxidases. Increased levels of ROS can chemically interact deleteriously with important biomolecules, causing protein carbonylation, DNA and RNA oxidation, DNA mutations [16] and lipid peroxidation, leading to organellar and cellular dysfunction and ultimately cell death. Oxidative stress in neurodegenerative diseases arises from an imbalance between the production of ROS and the level of antioxidants within the cell, leading to cell damage [8,17]. Previous methods to assess ROS levels are technically demanding [18] due to short half-lives and high reactivity, thus any sensor must function in a high spatial and temporal manner. Fortuitously, fluorescence imaging operates on a regime well suited to this [13] and is also often used in protein aggregation. Different classes of imaging probes such as nanomaterials and small molecule dyes have previously been used as fluorescence probes to study oxidative stress [19,20]. In this work, we demonstrate a new class of bifunctional-fluorescent small molecules that are capable of detecting both ROS and individual protein aggregates (figure 1*b–d*)

A major and abundant ROS that is heavily involved in cell signalling processes is hydrogen peroxide (H_2_O_2_). Additionally, the relatively high chemical stability of H_2_O_2_ results in significantly increased *in vivo*, steady state, concentrations in neurodegenerative disorders [21]. Aryl boronic acids and esters have been shown to effectively and selectively react with H_2_O_2_ and have thus been used broadly in drug delivery [22], as pro-chelators [23] and in H_2_O_2_ imaging [24–26]. We have, therefore, designed a novel strategy for the concomitant detection of both ROS and amyloid aggregates (figure 1*b*). We designed four fluorescent probes: BE01, BE02, mBE01 and mBE02 (figure 1*d*), all composed of a benzothiazole core for amyloid targeting and an aryl boronic ester-based H_2_O_2_-sensitive unit (figure 1*c*). These four imaging probes were evaluated for their ability to function as a bifunctional fluorescence probe using both exogenous H_2_O_2_ and aggregated recombinant *α*Syn protein, as a tractable model system.

## 2. Results and discussion

### 2.1. Synthesis

Beginning from known bromide X [27], palladium-catalysed borylation provided either BE01 or BE02 in a single step in good yields (figure 1*d*). Thereafter, treatment with trimethyloxonium tetrafluoroborate (Meerwein's salt) provided the corresponding *N*-methylbenzothiazolium dyes mBE01 and mBE02. Each compound is an easily handled solid that can be prepared in useful quantities (greater than 100 mg) and the compounds were confirmed with NMR spectroscopy (electronic supplementary material, figures S1 and S2).

### 2.2. Bulk photophysical properties

**Figure 2.**
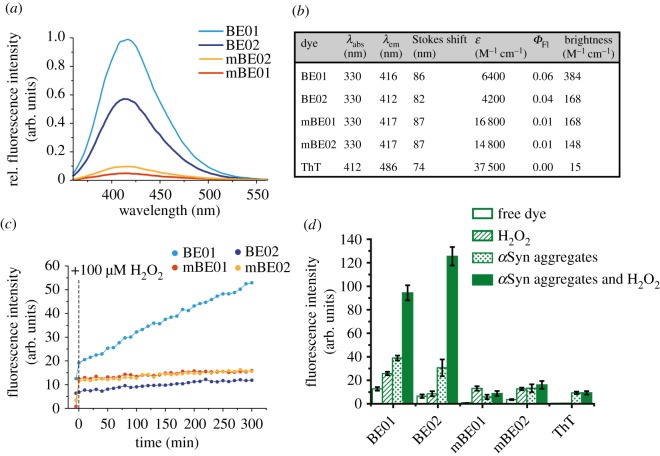
(*a*) Fluorescence spectra of the BE and mBE dyes free in solution at 5 µM. Intensities are shown relative to the maximum intensity of BE01. (*b*) Table of bulk photophysical properties (maximum absorption wavelength (*λ*_abs_), maximum emission wavelength (*λ*_em_), the Stokes shift (*λ*_em_ − *λ*_abs_), the brightness (*ε* × *Φ*_Fl_)). (*c*) Fluorescence versus time kinetics of the BE and mBE dyes upon the addition of H_2_O_2_. The first point represents the fluorescence intensity of the dyes prior to the addition of H_2_O_2_ (vertical dashed line). (*d*) Bar graph showing the fluorescence intensities of the BE, mBE and ThT dyes, in PBS buffer, with H_2_O_2_, with *α*Syn aggregates and with concomitant H_2_O_2_ and *α*Syn aggregates.

To initially characterize the efficacy of these new probes, their bulk photophysical properties were assessed in physiologically relevant conditions (PBS, pH 7.4) prior to the addition of either preformed, recombinant *α*Syn aggregates [28] or H_2_O_2_. All probes showed absorption maxima in the ultraviolet region at 330 nm, with emission maxima between 412 and 417 nm. The cationic mBE dyes displayed no shift in absorption or emission wavelength relative to the neutral dyes (figure 2*a*–*b*) suggesting that the methylation does not alter the extent of delocalization in the chromophore. The fluorescence intensities of the free dyes in this aqueous buffer environment were weak, with all dyes having a *Φ*_Fl_ < 0.1 (figure 2*b*). This observation was not wholly unexpected, due to both the electron withdrawing character of the boron and the non-emissive nature of the TICT state, like that observed with ThT [29,30]. The total integrated emission intensities (brightness) of the mBE dyes in solution were less than the neutral BE dyes, with higher *ε* values and lower *Φ*_Fl_, respectively. It is possible that the conformation in solution between the benzothiazole and phenyl-boronate rings in the methylated dyes remain somewhat, but not completely orthogonal (as in ThT) leading to low but detectable fluorescence at the bulk level [30].

### 2.3. Reactive oxygen species sensing

Next, the change in bulk fluorescence emission was assessed upon addition of H_2_O_2_ at a concentration realistic for cellular oxidative stress (100 µM) [31]. We observed an immediate increase in bulk fluorescence from all dyes upon the addition of H_2_O_2_ alone, (figure 2*c*) with mBE01 and mBE02 showing 16.0 and 3.6-fold increases and the neutral BE dyes showing more modest increases of 2.0-fold for BE01 and 1.3-fold for BE02 (figure 2*d*)_._ Furthermore, the kinetics could be followed, with all fluorescence traces increasing at varying rates in a linear regime over a 5 h period (figure 2*c*). As the oxidation reaction is predominantly irreversible, these sensors record the total ROS exposure to a system, rather than the equilibrium value. For BE01 we were able to detect H_2_O_2_ to a concentration greater than 1 µM and simultaneously showed significant changes in fluorescence intensity between 1 and 100 µM H_2_O_2_ (electronic supplementary material, figure S3). The oxidation of the electron withdrawing boronate ester to the electron donating hydroxyl was more significant with the cationic mBE dyes, hence the cationic dyes are more efficient sensors of H_2_O_2_ alone. As expected, no change in ThT fluorescence intensity was observed in response to H_2_O_2_ as no ester was present to be oxidized. To prove that the fluorescence changes seen are based on the oxidative cleavage of the boronic esters triggered by H_2_O_2_, identically prepared samples were analysed for their chemical composition via liquid chromatography mass spectrometry (LCMS, electronic supplementary material, table S1). These measurements confirmed that the change in fluorescence intensity arose from the conversion of the boronic ester into the hydroxyl functionality. By contrast, no significant change in the chemical composition of the dye solutions occurred over the same time frame without addition of H_2_O_2_ (electronic supplementary material, figure S4).

### 2.4. Aggregate sensing

**Figure 3.**
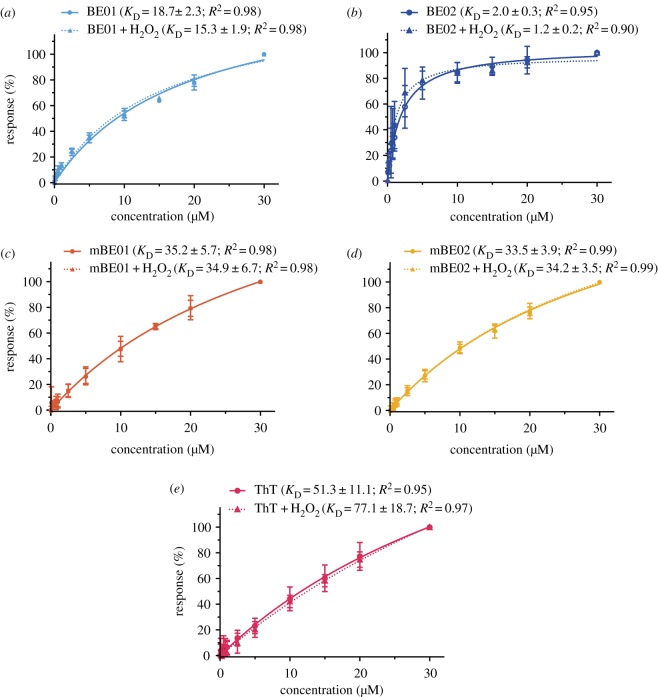
Equilibrium affinity analysis of *α*Syn interactions with (*a*) BE01, (*b*) BE02, (*c*) mBE01, (*d*) mBE02 and (*e*) ThT before (circular symbols, solid lines) and after (triangle symbols, dotted lines) incubation with 100 µM H_2_O_2_. The averaged (*n* ≥ 3), normalized SPR response upon injection of different concentrations of dye solution (30, 20, 15, 10, 5, 2.5, 1, 0.75, 0.5, 0.25, 0.1 µM) from independent measurements are shown. Dissociation constant (*K*_D_) obtained by nonlinear fitting.

As well as independent H_2_O_2_ sensing capabilities, all the dyes responded to the presence of approximately 10 µM preformed *α*Syn aggregates with a fluorescence turn-on. Both the BE and mBE dyes showed affinity for these *α*Syn aggregates (figure 3), the mechanism of which is assumed to be analogous to ThT [32]. The capability of the dyes to bind *α*Syn aggregates was confirmed with surface plasmon resonance (SPR) measurements, in which *α*Syn aggregates were covalently attached onto the chip surface via an amide bond. The binding of the different dyes to the aggregates was then investigated taking ThT as a reference compound. Compared with ThT (*K*_D_ = 51.2 ± 5.9 µM), all dyes showed improved binding to *α*Syn aggregates (figure 3). The non-methylated dyes (BE01 and BE02; *K*_D_^BE01^ = 18.7 ± 2.3 µM, *K*_D_^BE02^ = 2.0 ± 0.3 µM) outperformed their methylated counterparts (mBE01 and mBE02; *K*_D_^mBE01^ = 35.2 ± 5.7 µM; *K*_D_^BE02^ = 33.5 ± 3.9 µM), which is in agreement with the literature comparing methylated and non-methylated benzothiazole compounds [33]. The *K*_D_ of ThT has been reported to range from 0.033 to 64 µM [34]; we measured *K*_D_^ThT^ = 51.3 ± 11.1 µM, towards the upper end of this range. This is probably due to the fact that SPR is a surface bound method where fixed, heterogeneous aggregates [13] are continuously exposed to a solvent flow, whereas fluorescence-based measurements, most commonly reported [34], are carried out in solution. These data indicate that any change in fluorescence was not due to affinity changes but rather a modification of a fluorescence property of the dye itself. Upon the addition of *α*Syn aggregates, we saw an increase in fluorescence intensity of 3.1, 4.8, 7.1 and 3.8-fold for BE01, BE02, mBE01 and mBE02, respectively (figure 2*d*, electronic supplementary material, figure S5).

### 2.5. Bifunctional sensing

All four dyes have demonstrated capabilities of independent fluorescence sensing of either H_2_O_2_ or *α*Syn aggregates as the response to a chemical or physical modification of state. Excitingly, when H_2_O_2_ was combined with *α*Syn aggregates we saw a large increase in the total fluorescence emission of the BE01 and BE02 compounds (figure 2*d*, electronic supplementary material, figure S5). In BE01 while addition of either H_2_O_2_ or *α*Syn leads to a systematic increase in fluorescence of 2.0-fold and 3.3-fold, respectively, the addition of both together creates a ‘compounded' increase of 7.5-fold. Similarly, in the BE02 case, H_2_O_2_ by itself left the dye emission almost unchanged. However, an increase of 4.8-fold was observed upon the addition of *α*Syn aggregates, and the ‘compounded' increase was significantly (electronic supplementary material, table S2 and figure S6) higher at 19.6-fold. As expected, ThT showed no change in fluorescence in the presence of coincident H_2_O_2_ and *α*Syn aggregates. BE01 and BE02 showed significantly greater magnitudes of fluorescence intensity in comparison with mBE01 and mBE02, which showed no additional change in fluorescence in the bifunctional mode relative to *α*Syn aggregates alone. Taken together, these data support the idea that both BE01 and BE02 act as excellent bifunctional fluorescence sensors for concomitant detection of *α*Syn aggregates and H_2_O_2_ at the bulk level. As the non-methylated BE dyes gave integrated intensity greater to that of ThT, we explored whether they were sensitive enough to function at the single-aggregate level.

### 2.6. Single-aggregate and H_2_O_2_ fluorescence imaging

**Figure 4.**
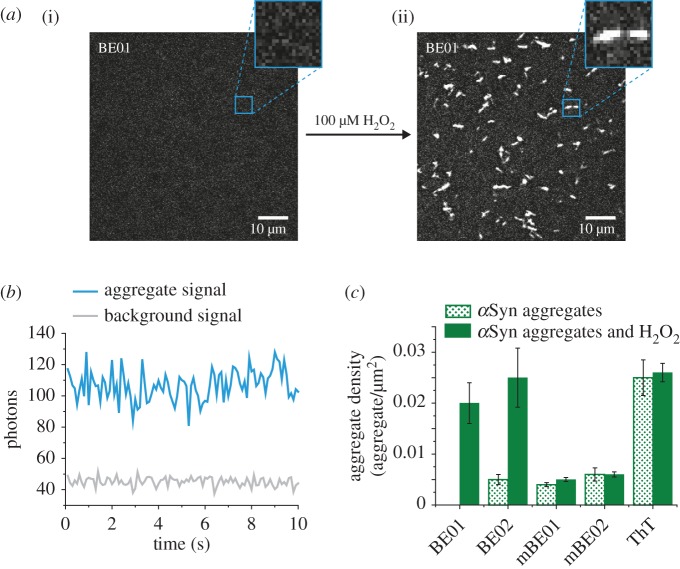
(*a*) Average intensity SAVE images of BE01 with αSyn aggregates before (i) and after (ii) addition of 100 µM H_2_O_2_. (*b*) An intensity versus time trace showing the photons detected from dyes on a single aggregate (blue) and background (grey) during a 10 s acquisition. (*c*) Density of single αSyn aggregate species detected by BE, mBE and ThT dyes before and after addition of 100 µM H_2_O_2_.

Next, the suitability of these probes was investigated for bifunctional *in vitro* studies at the single-aggregate level. We used a modified version of single-molecule total internal reflection fluorescence (TIRF) microscopy called single-aggregate visualization by enhancement (SAVE) imaging which we have previously demonstrated to detect and count single-aggregate species using ThT [13]. SAVE imaging was successfully applied to the new bifunctional probes, in all cases visualizing individual protein aggregates of *α*Syn on surfaces (figure 4*a*, electronic supplementary material, figure S7). This is exemplified in figure 4*a*, where BE01 displayed no detectable fluorescence in the presence of *α*Syn; however, with the addition of H_2_O_2_, the dye showed irreversible fluorescence activation (figure 4*a*) easily detecting single aggregates (SNR = approximately 15). Interactions of the BE and mBE dyes with *α*Syn showed fast exchange during the integration time of the detector, as with ThT (figure 4*b*) confirmed by constant, low fluorescence intensity over time and no apparent photobleaching [13]. Therefore, imaging for long periods of time would not be affected by the optical stability of these probes. To quantify this process, we determined the number of detectable aggregates, before and after addition of H_2_O_2_ and assumed constant surface coverage. No *α*Syn aggregate species were visualized by BE01 under this imaging mode prior to oxidation; however, upon activation with H_2_O_2_, the density of species detected increased to 0.020 ± 0.004 aggregates/µm^2^ (figure 4*c*). BE02 showed a fivefold increase in the density of detectable aggregates from 0.005 ± 0.001 to 0.025 ± 0.006 aggregates/µm^2^. As a control, ThT displayed no change in species density (0.025 ± 0.004 and 0.026 ± 002 aggregates/µm^2^). Interestingly, the densities of *α*Syn aggregates detected by oxidized BE01 and BE02 were similar to ThT despite the significantly higher fluorescence intensity of oxidized BE01 and BE02 with *α*Syn aggregates at the ensemble level. This suggests that both BE01 and BE02 are sensitive to similar species to ThT. There were very low densities of species detected by pre-oxidized mBE01 (0.0040 ± 0.0004 aggregates/µm^2^) and mBE02 (0.006 ± 0.001 aggregates/µm^2^) and negligible change in the density of species detected post-oxidation at 0.0051 ± 0.0004 aggregates/µm^2^ and 0.0060 ± 0.0005 aggregates/µm^2^ for mBE01 and mBE02 respectively. This agreed with the ensemble measurements and demonstrates the need for a neutral benzothiazole core for effective bifunctional sensing. The *N*-methylbenzothiazolium probes mBE01 and mBE02 can, however, be used in a limited capacity to measure either ROS activation or aggregate presence but do not show any advantage under exposure to both conditions. The limit of detection (LOD) of oxidized BE (BE-Ox) and mBE (mBE-Ox) probes to *α*Syn aggregates was determined using SAVE imaging (electronic supplementary material, figure S8). The LOD values are 4.58 ± 1.94 µM, 0.044 ± 0.005 nM and 2.40 ± 0.64 µM for BE-Ox, mBE-Ox and ThT, respectively, and were calculated by finding the first concentration at which the number of detected aggregates deviated significantly from the mean background (3 s.d.). The cellular biotoxicity of these probes was assessed for their potential use *in vivo* using a standard analytical cell-death assay (Trypan blue) and all probes both pre- and post-oxidation were found to not affect cell viability after a 24 h period (electronic supplementary material, figure S9) [35].

## 3. Conclusion

New methods for the detection of ROS and protein aggregates will be critical to advance our clinical understanding of diseases. Accordingly, we have described the design and successful application of a new class of bifunctional fluorescent probes for the independent or simultaneous detection of both *α*Syn aggregates and H_2_O_2_. We used existing strategies for independently detecting either β-sheet containing aggregates of amyloid or ROS and united them to create four dyes BE01, BE02, mBE01 and mBE02. The BE and mBE dyes are distinguished only by *N*-methylation of the benzothiazole core, which provides the corresponding benzothiazolium fluorophores. The increased positive charge appears to have a profound effect on the bifunctional capabilities of the mBE probes relative to the neutral BE congeners. Ensemble measurements showed the mBE probes were competent independent H_2_O_2_ or *α*Syn aggregate sensors but poor sensors of the two species simultaneously. This was further demonstrated with single-aggregate sensitivity. However, both BE01 and BE02 very successfully detected the simultaneous presence of both H_2_O_2_ and *α*Syn aggregates, providing large increases in ensemble fluorescence intensity and detected single aggregates. These tools may prove useful in cellular studies of neurodegeneration where it is desirable to study both processes simultaneously.

## 4. Experimental methods

### 4.1. Synthesis

#### 4.1.1. General considerations

All reactions were magnetically stirred and solvents purified under a dry argon atmosphere by passage through activated alumina and Q5 Grubbs apparatus. Work-up and purification procedures were conducted in air using reagent-grade solvents purchased from Sigma-Aldrich. Reactions were monitored by thin layer chromatography (TLC) using SiO_2_ on pre-coated glass plates, and a layer thickness of 0.25 mm. Compounds were visualized by UV light (254 and 366 nm) and a basic potassium permanganate stain (3 g KMnO_4_, 20 g K_2_CO_3_, 2.5 ml 10% aq. NaOH, in 400 ml DI H_2_O). Column chromatography was performed with silica gel 60 (35–70 µm). IR spectra were recorded on an Avatar 360-FT IR E.S.P. on a NaCl salt plate. Absorptions are reported as values in cm^−1^ followed by the relative intensity: *s*, strong; m, medium and *w*, weak. 1H NMR spectra were recorded at room temperature on a Varian I400, (400 MHz), I500 (500 MHz), I600 (600 MHz) or a Varian VXR400 (400 MHz) Fourier transform spectrometer using an internal deuterium lock. The chemical shift in ppm is quoted relative to the residual solvent peak of chloroform (*δ*H = 7.26, *δ*C = 77.05) as the internal standard, unless otherwise reported. Multiplicities are described as *s*, singlet; *d*, doublet; *t*, triplet; *m*, multiplet. Coupling constants are reported in Hz, and integration. Low mass spectrometry was carried out on an Agilent 1200 HPLC-6130 MSDAll using ESI, and mass to charge ratios (*m/z*) are reported as values in atomic mass units.

An overview of the synthetic route of the all four fluorescence probes is outlined in figure 1*d*.

#### 4.1.2. Preparation of 6-methoxy-2-(4-(4,4,5,5-tetramethyl-1,3,2-dioxaborolan-2-yl)phenyl)benzo[*d*]thiazole, BE01

2-(4-Bromophenyl)-6-methoxybenzo[*d*]thiazole (960 mg, 3 mmol, 1 equiv.), bis(pinacolato)diboron (768 mg, 3.03 mmol, 1.01 equiv.), and KOAc (879 mg, 9 mmol, 3 equiv.) were added to a dry round-bottomed flask and 1,4-dioxane (15 ml, 0.2 M) added under an atmosphere of N_2_. The solution was then degassed for 30 min by bubbling through N_2_. Pd(dppf)Cl_2_·DCM (72 mg, 0.09 mmol, 3 mol%) was then added and the vessel purged with N_2_ before being heated to 100°C for 18 h. The reaction mixture was then allowed to cool to room temperature and filtered through celite, washing with Et_2_O (20 ml). The filtrate was concentrated under reduced pressure and the residue was dissolved in Et_2_O (20 ml) and washed with H_2_O (20 ml) and brine (20 ml). The organic phase was dried over Na_2_SO_4_, filtered and concentrated under reduced pressure. The residue was purified by flash column chromatography (silica gel, 10% Et_2_O in petroleum ether) to give the *product* as a yellow solid (691 mg, 63%).

*υ*_max_ (solid): 2969, 2924, 1599, 1488, 1354, 1264, 1139, 1088, 823, 651 cm^−1^.

^1^H NMR (CDCl_3_, 400 MHz): *δ* 8.04 (d, *J* = 7.9 Hz, 2H), 7.96 (d, *J* = 9.0 Hz, 1H), 7.91 (d, *J* = 7.8 Hz, 2H), 7.35 (s, 1H), 7.16–7.02 (m, 1H), 3.89 (s, 3H), 1.37 (s, 12H).

^13^C NMR (CDCl_3_, 101 MHz): *δ* 165.6, 158.0, 148.9, 136.7, 136.1, 135.5, 126.5, 124.0, 115.9, 104.3, 84.2, 56.0, 25.0. Carbon bearing boron not observed.

HRMS: exact mass calculated for [M]^+^ (C_20_H_22_BNO_3_S) requires *m/z* 367.1408, found *m/z* 367.1391.

#### 4.1.3. Preparation of 2-(4-(5,5-dimethyl-1,3,2-dioxaborinan-2-yl)phenyl)-6-methoxybenzo[*d*]thiazole, BE02

2-(4-Bromophenyl)-6-methoxybenzo[*d*]thiazole (320 mg, 1 mmol, 1 equiv.), bis(neopentyl glycolato)diboron (226 mg, 1.01 mmol, 1.01 equiv.), and KOAc (293 mg, 3 mmol, 3 equiv.) were added to a dry round-bottomed flask and 1,4-dioxane (5 ml, 0.2 M) added under an atmosphere of N_2_. The solution was then degassed for 30 min by bubbling through N_2_. Pd(dppf)Cl_2_·DCM (72 mg, 0.09 mmol, 3 mol%) was then added and the vessel purged with N_2_ before being heated to 100°C for 18 h. The reaction mixture was then allowed to cool to room temperature and filtered through celite, washing with Et_2_O (20 ml). The filtrate was concentrated under reduced pressure and the residue was dissolved in Et_2_O (20 ml) and washed with H_2_O (20 ml) and brine (20 ml). The organic phase was dried over Na_2_SO_4_, filtered and concentrated under reduced pressure. The residue was purified by flash column chromatography (silica gel, 10–40% Et_2_O in petroleum ether) to give the *product* as a yellow solid (198 mg, 56%).

*υ*_max_ (solid): 2960, 2931, 1598, 1489, 1475, 1313, 1305, 1269, 1247, 1227, 1129, 818, 643 cm^−1^.

^1^H NMR (CDCl_3_, 400 MHz): *δ* 8.02 (d, *J* = 8.3 Hz, 2H), 7.96 (d, *J* = 9.0 Hz, 1H), 7.90 (d, *J* = 8.2 Hz, 2H), 7.36 (d, *J* = 2.5 Hz, 1H), 7.09 (dd, *J* = 9.0, 2.6 Hz, 1H), 3.90 (s, 3H), 3.80 (s, 4H), 1.05 (s, 6H).

^13^C NMR (CDCl_3_, 101 MHz): *δ* 165.4, 157.5, 148.4, 136.2, 135.2, 134.2, 125.0, 123.5, 115.4, 103.8, 72.1, 55.5, 31.6, 21.6. Carbon bearing boron not observed.

HRMS: exact mass calculated for [M]^+^ (C_19_H_20_BNO_3_S) requires *m/z* 353.1251, found *m/z* 353.1257.

#### 4.1.4. 6-Methoxy-3-methyl-2-(4-(4,4,5,5-tetramethyl-1,3,2-dioxaborolan-2-yl)phenyl)benzo[*d*]thiazol-3-ium tetrafluoroborate, BE02

6-Methoxy-2-(4-(4,4,5,5-tetramethyl-1,3,2-dioxaborolan-2-yl)phenyl)benzo[*d*]thiazole (367 mg, 1 mmol, 1 equiv.) and trimethyloxonium tetrafluoroborate (148 mg, 1 mmol, 1 equiv.) were added to a dry round-bottomed flask and DCM (4 ml, 0.25 M) added under an atmosphere of N_2_. The reaction mixture was stirred at room temperature for 18 h before the addition of Et_2_O (20 ml), which formed a yellow precipitate. The solid was collected by filtration and washed with Et_2_O (2 × 20 ml) before being dried under air to give the *product* as a yellow solid (362 mg, 77%).

*υ*_max_ (solid): 2980, 1597, 1400, 1366, 1278, 1144, 1059, 1018, 855, 825, 654 cm^−1^.

^1^H NMR (DMSO-*d*_6_, 400 MHz): *δ* 8.33 (d, *J* = 9.3 Hz, 1H), 8.09 (d, *J* = 2.6 Hz, 1H), 8.01 (d, *J* = 8.1 Hz, 2H), 7.94 (d, *J* = 8.2 Hz, 2H), 7.59 (dd, *J* = 9.3, 2.6 Hz, 1H), 4.20 (s, 3H), 3.95 (s, 3H), 1.34 (s, 12H).

^13^C NMR (DMSO-*d*_6_, 101 MHz): *δ* 170.9, 159.5, 136.8, 135.2, 131.7, 129.8, 127.9, 119.5, 118.7, 106.3, 84.4, 56.3, 38.1, 24.7. Carbon bearing boron not observed.

HRMS: exact mass calculated for [M-BF_4_]^+^ (C_21_H_25_BNO_3_S^+^) requires *m/z* 382.1652, found *m/z* 382.1665.

#### 4.1.5. 2-(4-(5,5-Dimethyl-1,3,2-dioxaborinan-2-yl)phenyl)-6-methoxy-3-methylbenzo[*d*]thiazol-3-ium tetrafluoroborate mBE02

2-(4-(5,5-Dimethyl-1,3,2-dioxaborinan-2-yl)phenyl)-6-methoxybenzo[*d*]thiazole (150 mg, 0.42 mmol, 1 equiv.) and trimethyloxonium tetrafluoroborate (62 mg, 0.42 mmol, 1 equiv.) were added to a dry round-bottomed flask and DCM (1.7 ml, 0.25 M) added under an atmosphere of N_2_. The reaction mixture was stirred at room temperature for 18 h before the addition of Et_2_O (10 ml), which formed a yellow precipitate. The solid was collected by filtration and washed with Et_2_O (2 × 10 ml) before being dried under air to give the *product* as a yellow solid (156 mg, 82%).

*υ*_max_ (solid): 1599, 1481, 1424, 1340, 1316, 1307, 1253, 1132, 1055, 1036 cm^−1^.

^1^H NMR (DMSO-*d*_6_, 400 MHz): *δ* 8.33 (d, *J* = 9.3 Hz, 1H), 8.09 (d, *J* = 2.5 Hz, 1H), 8.02 (d, *J* = 7.8 Hz, 2H), 7.91 (d, *J* = 7.9 Hz, 2H), 7.59 (dd, *J* = 9.3, 2.5 Hz, 1H), 4.21 (s, 3H), 3.95 (s, 3H), 3.83 (s, 4H), 0.99 (s, 6H).

^13^C NMR (DMSO-*d*_6_, 101 MHz): *δ* 171.1, 159.4, 136.8, 134.3, 131.6, 129.5, 127.3, 119.4, 118.7, 106.3, 71.6, 56.3, 38.1, 31.5, 21.2. Carbon bearing boron not observed.

HRMS: exact mass calculated for [M-BF_4_]^+^ (C_20_H_23_BNO_3_S^+^) requires *m/z* 368.1496, found *m/z* 368.1519.

#### 4.1.6. Preparation of dye solutions for ensemble fluorescence measurements

Stock solutions of BE, mBE compounds and ThT (Sigma-Aldrich, T3516) were prepared by dissolving the solid form into dimethyl sulfoxide (DMSO, Sigma-Aldrich, 276 855) to a concentration of 1 mM. These stock solutions were stored in the dark at −80°C. The stock solutions were subsequently diluted into filtered (0.02 µm syringe filter, Whatman, 6809–1102) PBS (pH 7.4) to a concentration of 25 µM. The diluted dye solutions were then filtered (0.02 µm syringe filter, Whatman, 6809–1102). The diluted solutions were stored in the dark at 4°C for a maximum of a week after preparation.

#### 4.1.7. Preparation of *α*Syn aggregates

Monomeric wild-type *α*Syn was purified from *Escherichia coli* as previously described in [36]. To generate amyloid fibrils a solution of *α*Syn (70 µM) in PBS buffer (pH 7.4) with 0.01% NaN_3_ to prevent bacterial growth was incubated in the dark at 37°C with constant agitation (200 rpm) for several months before use.

## 5. Bulk fluorescence measurements

### 5.1. General considerations

H_2_O_2_ was prepared by diluting a stock solution (9.7 M, VWR) into filtered ultra-pure, 18.2 MΩ cm water (Merck, SIMSV00WW) (0.2 µm syringe filter, Whatman, 6780–1302) to 10 mM. Samples were placed into a quartz fluorescence cuvette (Hellma Analytics, 3 × 3 mm) and bulk fluorescence spectra were recorded using a fluorescence spectrophotometer (Cary Eclipse, Varian). Fluorescence spectra of BE, mBE dyes and ThT (5 µM) were recorded free in PBS (pH 7.4), in the presence of H_2_O_2_ (100 µM), in the presence of *α*Syn aggregates (approx. 10 µM) and in the presence of both H_2_O_2_ and *α*Syn aggregates simultaneously at excitation maxima. The error bars (figure 2*d*) represent standard deviations from experiments with *n* = 3 separately prepared dye samples.

To assess the ensemble fluorescence response of the dyes to H_2_O_2_ the dyes were incubated with H_2_O_2_ and the fluorescence intensity recorded over 5 h at 20 min intervals. The curves have a positive gradient (BE01 = 0.112 ± 0.002 min^−1^, BE02 = 0.017 min^−1^_,_ mBE01 = 0.011 min^−1^_,_ mBE02 = 0.015 min^−1^)(figure 2*c*). Fluorescence emission was recorded instantly following incubation of the dyes with *α*Syn aggregates. The dyes (25 µM) were then incubated with H_2_O_2_ (100 µM) for 60 min. Following this incubation period the dyes were diluted to 5 µM and the emission spectra were recorded with *α*Syn aggregates. Spectra of the free dyes in PBS were also recorded at appropriate excitation wavelengths for calculation of *Φ*_Fl_ relative to a standard [37].

### 5.2. Determination of ensemble photophysical properties

Several photophysical quantities were determined from ensemble absorption and emission data, using techniques previously described [38]. These included absorption maxima (*λ*_Abs_), emission maxima (*λ*_Em_) Stokes shift, molar extinction coefficient (*ε*) and fluorescence quantum yield (*Φ*_Fl_). Stokes shifts were calculated from the difference of *λ*_Em_ and *λ*_Abs_. *ε* was determined using the Beer–Lambert law with solutions of known concentrations. *Φ*_Fl_ of the BE and mBE dyes in PBS were referenced against quinine sulfate (Sigma-Aldrich, Q0132) in 0.1 M H_2_SO_4_ (*Φ*_Fl_ = 0.5) [37], ThT was referenced against rhodamine 101 (Sigma-Aldrich, 83694) in ethanol (*Φ*_Fl_ = 1) [39] and corrections were made for discrepancies in absorbance and solvent refractive index.

### 5.3. Surface plasmon resonance measurements

SPR studies were performed at 25°C using a Biacore T200 in HBS-EP^+^ buffer (10 mM HEPES, 150 mM sodium chloride, 3 mM EDTA and 0.05% v/v surfactant P20 in ultra-pure, 18.2 MΩ cm water, Merck, SIMSV00WW) and filtered through a vacuum-driven filter system (0.22 µm, Merck) containing DMSO (5% v/v). *α*Syn aggregates, prepared as described previously, were covalently coupled to a research grade CM5 sensor chip (BIAcore) via primary amino functionalities of the fibrils using the amine coupling kit provided by BIAcore. For the coupling step, *α*Syn aggregates were injected at a concentration of 2.8 µM in sodium acetate buffer (10 mM, pH 4.0) at a flow rate of 10 µl min^−1^ for 12 min. Coupling levels ranged from 2146 to 11 350 RU. For reference subtraction, a second flow cell was treated identically including all immobilization chemistries but without *α*Syn aggregates.

The dye samples were prepared by diluting the dye stock solutions (10 mM) down to obtain a 30 µM solution containing 5% DMSO. To prepare the desired concentration series (30, 20, 15, 10, 5, 2.5, 1, 0.75, 0.5, 0.25, 0.1 µM), the 30 µM sample was further diluted using the assay running buffer (HBS-EP^+^ + 5% DMSO). To acquire the binding curves of the oxidized dyes, the 30 µM sample was incubated for 1 h with 100 µM H_2_O_2_ before further dilution.

At the beginning and at the end of each measurement set a solvent correction assay was performed to correct for the DMSO content in the samples. After stabilization of the baseline, response units for each dye sample were acquired by using a flow rate of 30 µl min^−1^ for 60 s. Dissociation time was set to 400 s. No further regeneration step was needed. Each dye concentration series was measured in a randomized order and the 10 µM sample was measured a second time after the whole concentration series of the dye to prove consistency. In between the measurements of different dyes a 5 µM ThT sample was measured as control sample. The whole SPR measurement set for each dye was repeated at least three times on different flow cells with fresh immobilized *α*Syn and new prepared concentration series. All data are solvent corrected and reference subtracted. The obtained binding levels from each concentration series were baseline corrected, normalized and the normalized values averaged over the replicates (*n* ≥ 3). Error bars represent twice the standard deviations of the binding level values between the replicates. The averaged values were then plotted as a function of concentration (figure 3). *K*_D_ values were obtained by nonlinear curve fitting to these values using the equation *Y* = *B*_max_ × *X*/(*K*_D_ + *X*), where *Y* is the response, *X* the concentration, *B*_max_ the maximum specific binding response (same unit as *Y*) and *K*_D_ is the equilibrium binding constant (in the same units as X). The obtained fitting parameters are stated in electronic supplementary material, table S2.

### 5.4. Preparation for single-aggregate visualization by enhancement fluorescence imaging

Glass cover-slides for SAVE fluorescence imaging were cleaned for 1 h using an argon plasma (PDC-002, Harrick Plasma) and the slide surface incubated with poly-l-lysine (PLK) for 30 min as previously described [13]. Working solutions of dye (10 µM–1 pM) and *α*Syn (0.5 µM) in filtered (0.2 µm syringe filter, Whatman, 6780–1302) PBS (pH 7.4) were prepared 5 min prior to imaging. The working solution (50 µl) was added to the clean, PLK coated cover-slide and incubated for 2 min. The slide was then washed twice with PBS and imaged. H_2_O_2_ (100 µM) was then added directly to the cover-slide and the sample imaged again.

### 5.5. SAVE fluorescence imaging

Fluorescence imaging experiments were carried out with a bespoke inverted microscope (Nikon, Eclipse, TE2000-U) set-up optimized for TIRF measurements. Excitation of BE, mBE compounds and ThT in the presence of *α*Syn aggregates were performed with a collimated 405 nm laser (Oxxius, Laserboxx, LBX-405-50-CIR-PP) which was aligned parallel to the optical axis and directed into an oil immersion objective lens (1.45 NA, 60×, Plan Apo, TIRF, Nikon) above the critical angle to ensure TIR at the coverslip–sample (glass/water) interface. Fluorescence emission was also collected by the same objective and selected by the presence of a dichroic (Di01-R405/488/561/635, Semrock) and subsequently passed through appropriate emission filters (BLP01-488R-25, Semrock). Images were then recorded by an EMCCD camera (Evolve delta 512, Photometrics) with an electron multiplication gain of 250 running in frame transfer, clear presequence mode. Each pixel on the image was 275 nm. Images were recorded at 100 ms exposure for 200 frames using automation through Micro-manager software [40].

### 5.6. Single-aggregate fluorescence image analysis

Images were analysed with a bespoke ImageJ [41] macro. Image stacks of 200 frames taken as 100 ms per frame of the BE, mBE dyes and ThT with *α*Syn aggregates and either in the presence or absence of H_2_O_2_ were compressed in time to create single frame images representing the average pixel intensities. Pixels of intensity above background were determined with a fixed threshold and *α*Syn aggregate species in each field of view identified and counted. The density of species was determined from the division of the number of species by the area of the field of view (4726 µm^2^). Five fields of view in the presence of H_2_O_2_ and five in the absence of H_2_O_2_ were analysed for each dye. Error bars represent the standard deviations of the species density between the five fields of view.

### 5.7. Cell-viability assay

HEK293 cells were maintained in high-glucose DMEM supplemented with 10% (vol/vol) FBS and 1% penicillin and streptomycin at 37°C and 5% CO_2_. Trypan blue exclusion was used to determine cell viability. Briefly, HEK293 cells were seeded at 80% confluence in six-well plates and treated with 10 µM BE01, BE02, mBE01, mBE02, BE-Ox and mBE-Ox separately. Cell viabilities were sequentially tested at 3, 12 (data not shown) and 24 h after the treatment. The total cell population and trypan blue stained cells were counted under a microscope. Healthy cell cultures were considered to be more than 85% viable cells as determined by the following equation:

$$\text{viable cells}=\left(1-\left(\frac{\text{number of blue cells}}{\text{total number of cells}}\right)\right)\times 100$$

## Supplementary Material

SI.docx

## Data accessibility

This article has no additional data.

## References

[1] Chiti F, Dobson CM 2006 Protein misfolding, functional amyloid, and human disease. Annu. Rev. Biochem. 75, 333–366. (doi:10.1146/annurev.biochem.75.101304.123901) 16756495 10.1146/annurev.biochem.75.101304.123901

[2] Kumar V, Sami N, Kashav T, Islam A, Ahmad F, Hassan MI 2016 Protein aggregation and neurodegenerative diseases: from theory to therapy. Eur. J. Med. Chem. 124, 1105–1120. (doi:10.1016/j.ejmech.2016.07.054) 27486076 10.1016/j.ejmech.2016.07.054

[3] Ingelsson M, Fukumoto H, Newell KL, Growdon JH, Hedley-Whyte ET, Frosch MP, Albert MS, Hyman BT, Irizarry MC 2004 Early amyloid accumulation and progressive synaptic loss, gliosis and tangle formation in the Alzheimer's disease brain. Neurology 62, 925–931. (doi:10.1212/01.WNL.0000115115.98960.37) 15037694 10.1212/01.wnl.0000115115.98960.37

[4] Fearnley J, Lees A 1991 Ageing and Parkinson's disease: substantia nigra regional selectivity. Brain 114, 2283–2301. (doi:10.1093/brain/114.5.2283) 1933245 10.1093/brain/114.5.2283

[5] Gandhi S, Abramov AY 2012 Mechanism of oxidative stress in neurodegeneration. Oxid. Med. Cell. Longev. 2012, 1–11. (doi:10.1155/2012/428010) 10.1155/2012/428010 PMC3362933 22685618

[6] Deas E et al. 2016 Alpha-synuclein oligomers interact with metal ions to induce oxidative stress and neuronal death in Parkinson's disease. Antioxid. Redox Signal. 24, 376–391. (doi:10.1089/ars.2015.6343) 26564470 10.1089/ars.2015.6343 PMC4999647

[7] Cremades N et al. 2012 Direct observation of the interconversion of normal and toxic forms of α-synuclein. Cell 149, 1048–1059. (doi:10.1016/j.cell.2012.03.037) 22632969 10.1016/j.cell.2012.03.037 PMC3383996

[8] Lin MT, Beal MF 2006 Mitochondrial dysfunction and oxidative stress in neurodegenerative diseases. Nature 443, 787–795. (doi:10.1038/nature05292) 17051205 10.1038/nature05292

[9] Vilar M, Chou H-T, Lührs T, Maji SK, Riek-Loher D, Verel R, Manning G, Stahlberg H, Riek R 2008 The fold of alpha-synuclein fibrils. Proc. Natl Acad. Sci. USA 105, 8637–8642. (doi:10.1073/pnas.0712179105) 18550842 10.1073/pnas.0712179105 PMC2438424

[10] Sunde M, Blake C 1998 From the globular to the fibrous state: protein structure and structural conversion in amyloid formation. Q Rev. Biophys. 31, 1–39. (doi:10.1017/S0033583598003400) 9717197 10.1017/s0033583598003400

[11] Stefani M 2004 Protein misfolding and aggregation: new examples in medicine and biology of the dark side of the protein world. Biochim. Biophys. Acta Mol. Basis Dis. 1739, 5–25. (doi:10.1016/j.bbadis.2004.08.004) 10.1016/j.bbadis.2004.08.004 15607113

[12] Biancalana M, Koide S 2010 Molecular mechanism of thioflavin-T binding to amyloid fibrils. Biochim. Biophys. Acta Proteins Proteomics. Jul [cited 2014 Aug 5]; 1804, 1405–1412. (doi:10.1016/j.bbapap.2010.04.001) 10.1016/j.bbapap.2010.04.001 PMC2880406 20399286

[13] Horrocks MH et al. 2016 Single-molecule imaging of individual amyloid protein aggregates in human biofluids. ACS Chem. Neurosci. 7, 399–406. (doi:10.1021/acschemneuro.5b00324) 26800462 10.1021/acschemneuro.5b00324 PMC4800427

[14] Flagmeier P et al. 2017 Ultrasensitive measurement of Ca^2+^ influx into lipid vesicles induced by protein aggregates. Angew. Chemie Int. Ed. 56, 7750–7754. (doi:10.1002/anie.201700966) 10.1002/anie.201700966 PMC5615231 28474754

[15] Hayyan M, Hashim MA, Alnashef IM 2016 Superoxide ion: generation and chemical implications. Chem. Rev. 116, 3029–3085. (doi:10.1021/acs.chemrev.5b00407) 26875845 10.1021/acs.chemrev.5b00407

[16] Czarny P, Wigner P, Galecki P, Sliwinski T 2018 The interplay between inflammation, oxidative stress, DNA damage, DNA repair and mitochondrial dysfunction in depression. Prog. Neuro. Psychopharmacol. Biol. Psychiatry 80, 309–321. (doi:10.1016/j.pnpbp.2017.06.036) 10.1016/j.pnpbp.2017.06.036 28669580

[17] Andersen JK 2004 Oxidative stress in neurodegeneration: cause or consequence? Nat. Rev. Neurosci. 10, S18–S25. (doi:10.1038/nrn1434) 10.1038/nrn1434 15298006

[18] Sharma P, Jha AB, Dubey RS, Pessarakli M 2012 Reactive oxygen species, oxidative damage, and antioxidative defense mechanism in plants under stressful conditions. J. Bot. 2012, 1–26. (doi:10.1155/2012/217037)

[19] Guo Z, Park S, Yoon J, Shin I 2014 Recent progress in the development of near-infrared fluorescent probes for bioimaging applications. Chem. Soc. Rev. 43, 16–29. (doi:10.1039/C3CS60271K) 24052190 10.1039/c3cs60271k

[20] Zhang X, Kong R, Tan Q, Qu F, Qu F 2017 A label-free fluorescence turn-on assay for glutathione detection by using MnO_2_ nanosheets assisted aggregation-induced emission-silica nanospheres. Talanta 169, 1–7. (doi:10.1016/j.talanta.2017.03.050) 28411797 10.1016/j.talanta.2017.03.050

[21] Szatrowski TP, Nathan CF 1991 Production of large amounts of hydrogen peroxide by human tumor cells. Cancer Res. 51, 794–798. 1846317

[22] Kuang Y, Balakrishnan K, Gandhi V, Peng X 2011 Hydrogen peroxide inducible DNA cross-linking agents: targeted anticancer prodrugs. J. Am. Chem. Soc. 133, 19 278–19 281. (doi:10.1021/ja2073824) 10.1021/ja2073824 PMC3265938 22035519

[23] Charkoudian LK, Pham DM, Franz KJ 2006 A pro-chelator triggered by hydrogen peroxide inhibits iron-promoted hydroxyl radical formation. J. Am. Chem. Soc. 128, 12 424–12 425. (doi:10.1021/ja064806w) 16984186 10.1021/ja064806w

[24] Dickinson BC, Huynh C, Chang CJ 2010 A palette of fluorescent probes with varying emission colors for imaging hydrogen peroxide signaling in living cells. J. Am. Chem. Soc. 132, 5906–5915. (doi:10.1021/ja1014103) 20361787 10.1021/ja1014103 PMC2862989

[25] Carroll V, Michel BW, Blecha J, Vanbrocklin H, Keshari K, Wilson D, Chang CJ 2014 A boronate-caged [^18^F]FLT probe for hydrogen peroxide detection using positron emission tomography. J. Am. Chem. Soc. 136, 14 742–14 745. (doi:10.1021/ja509198w) 10.1021/ja509198w PMC4210116 25310369

[26] Kim D, Kim G, Nam S-J, Yin J, Yoon J 2015 Visualization of endogenous and exogenous hydrogen peroxide using a lysosome-targetable fluorescent probe. Sci. Rep. 5, 8488 (doi:10.1038/srep08488) 25684681 10.1038/srep08488 PMC4329546

[27] Yang P, Wang R, Wu H, Du Z, Fu Y 2017 Pd-catalyzed C–H arylation of benzothiazoles with diaryliodonium salt: one-pot synthesis of 2-arylbenzothiazoles. Asian J. Org. Chem. 6, 184–188. (doi:10.1002/ajoc.201600514)

[28] Horrocks MH et al. 2015 Fast flow microfluidics and single-molecule fluorescence for the rapid characterization of β-synuclein oligomers. Anal. Chem. 87, 8818–8826. (doi:10.1021/acs.analchem.5b01811) 26258431 10.1021/acs.analchem.5b01811

[29] Stsiapura VI, Maskevich AA, Kuzmitsky VA, Turoverov KK, Kuznetsova IM 2007 Computational study of thioflavin T torsional relaxation in the excited state. J. Phys. Chem. A 111, 4829–4835. (doi:10.1021/jp070590o) 17497763 10.1021/jp070590o

[30] Maskevich AA, Stsiapura VI, Kuzmitsky VA, Kuznetsova IM, Povarova OI, Uversky VN, Turoverov KK 2007 Spectral properties of thioflavin T in solvents with different dielectric properties and in a fibril-incorporated form. J. Proteome Res. 6, 1392–1401. (doi:10.1021/pr0605567) 17305383 10.1021/pr0605567

[31] Halliwell B, Celement MV, Lee HL 2000 Hydrogen peroxide in the human body. FEBS Lett. 486, 10–13. (doi:10.1016/S0014-5793(00)02197-9) 11108833 10.1016/s0014-5793(00)02197-9

[32] Biancalana M, Makabe K, Koide A, Koide S 2009 Molecular mechanism of thioflavin-T binding to the surface of β-rich peptide self-assemblies. J. Mol. Biol. 385, 1052–1063. (doi:10.1016/j.jmb.2008.11.006) 19038267 10.1016/j.jmb.2008.11.006 PMC2664162

[33] Wu C, Bowers MT, Shea JE 2011 On the origin of the stronger binding of PIB over thioflavin T to protofibrils of the Alzheimer amyloid-β peptide: a molecular dynamics study. Biophys. J. 100, 1316–1324. (doi:10.1016/j.bpj.2011.01.058) 21354405 10.1016/j.bpj.2011.01.058 PMC3043208

[34] Groenning M 2010 Binding mode of thioflavin T and other molecular probes in the context of amyloid fibrils-current status. J. Chem. Biol. 3, 1–18. (doi:10.1007/s12154-009-0027-5) 19693614 10.1007/s12154-009-0027-5 PMC2816742

[35] Louis KS, Siegel AC 2011 Cell viability analysis using trypan blue: manual and automated methods. Methods Mol. Biol. 740, 7–12. (doi:10.1007/978-1-61779-108-6_2) 21468962 10.1007/978-1-61779-108-6_2

[36] Hoyer W, Antony T, Cherny D, Heim G, Jovin TM, Subramaniam V 2002 Dependence of a -synuclein aggregate morphology on solution conditions. J. Mol. Biol. 2836, 383–393. (doi:10.1016/S0022-2836(02)00775-1) 10.1016/s0022-2836(02)00775-1 12217698

[37] Lakowicz JR 2006 Principles of fluorescence spectroscopy. New York, NY: Springer Science & Business Media.

[38] Lord SJ, Lu Z, Wang H, Willets KA, Schuck PJ, Lee HLD, Nishimura SY, Twieg RJ, Moerner WE 2007 Photophysical properties of acene DCDHF fluorophores: Long-wavelength single-molecule emitters designed for cellular imaging. J. Phys. Chem. A 111, 8934–8941. (doi:10.1021/jp0712598) 17718454 10.1021/jp0712598 PMC2678804

[39] Magde D, Rojas GE, Seybold PG 1999 Solvent dependence of the fluorescence lifetimes of xanthene dyes. Photochem. Photobiol. 70, 737–744. (doi:10.1111/j.1751-1097.1999.tb08277.x)

[40] Edelstein AD, Tsuchida MA, Amodaj N, Pinkard H, Vale RD, Stuurman N 2014 Advanced methods of microscope control using μManager software. J. Biol. Methods 1, 10 (doi:10.14440/jbm.2014.36) 10.14440/jbm.2014.36 PMC4297649 25606571

[41] Schindelin J et al. 2012 Fiji: an open-source platform for biological-image analysis. Nat. Methods 9, 676–682. (doi:10.1038/nmeth.2019) 22743772 10.1038/nmeth.2019 PMC3855844

