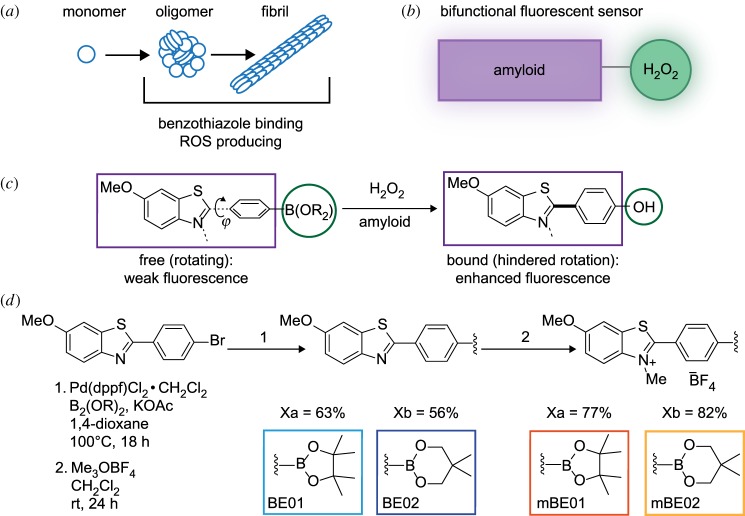# Correction to ‘Bifunctional fluorescent probes for detection of amyloid aggregates and reactive oxygen species’

**DOI:** 10.1098/rsos.180308

**Published:** 2018-03-28

**Authors:** Lisa-Maria Needham, Judith Weber, James W. B. Fyfe, Omaru M. Kabia, Dung T. Do, Ewa Klimont, Yu Zhang, Margarida Rodrigues, Christopher M. Dobson, Sonia Gandhi, Sarah E. Bohndiek, Thomas N. Snaddon, Steven F. Lee

*R. Soc. open sci.* **5**, 171399. (Published 7 February 2018). (doi:10.1098/rsos.171399)

In the published paper, Sonia Gandhi's name is misspelled as ‘Sonia Ghandi’. The corrected author list is provided above.

Figure 1*c* is presented incorrectly in the published paper. The letter ‘j’ underneath the structure should be a Greek letter phi. The correct figure is shown below.

**Figure:**