# Supplementary material for: Bifunctional fluorescent probes for detection of amyloid aggregates and reactive oxygen species
Source: R Soc Open Sci. 2018 Feb 7;5(2):171399. doi: 10.1098/rsos.171399 (PMC5830749; doi:10.1098/rsos.171399)
Supplement: SI.docx [file rsos171399supp1.docx]

Supplementary Information

**Bifunctional fluorescent probes for detection of amyloid aggregates and reactive oxygen species**

Lisa-Maria Needham*^,1^, Judith Weber*^,2,3^, James W. B. Fyfe^4^, Omaru M. Kabia^4^, Dung T. Do^4^, Ewa Klimont^1^, Yu Zhang^1^, Margarida Rodrigues^1^, Christopher M. Dobson^1^, Sonia Ghandi^5^, Sarah E. Bohndiek^#,2,3^, Thomas N. Snaddon^#,4^, Steven F. Lee^#,1^.


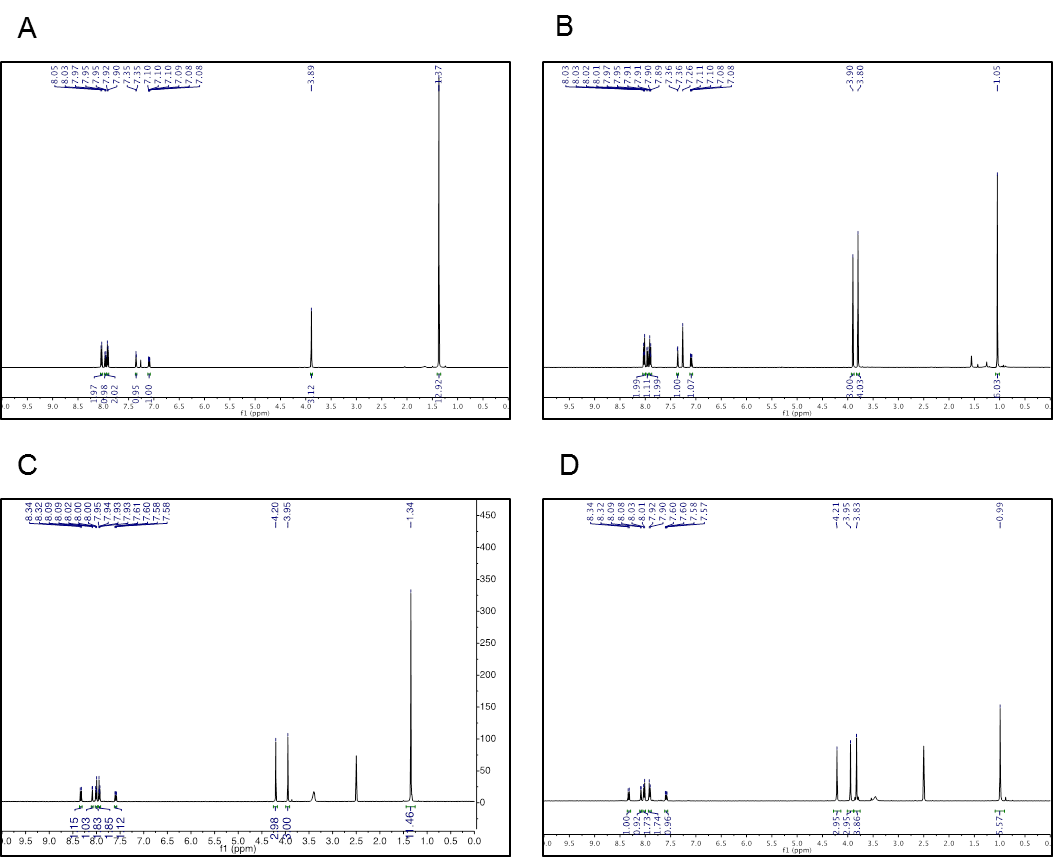


**Supplementary Figure 1.** ^1^H NMR spectra of **A)** BE01 **B)** BE02 **C)** mBE01 and **D)** mBE02.


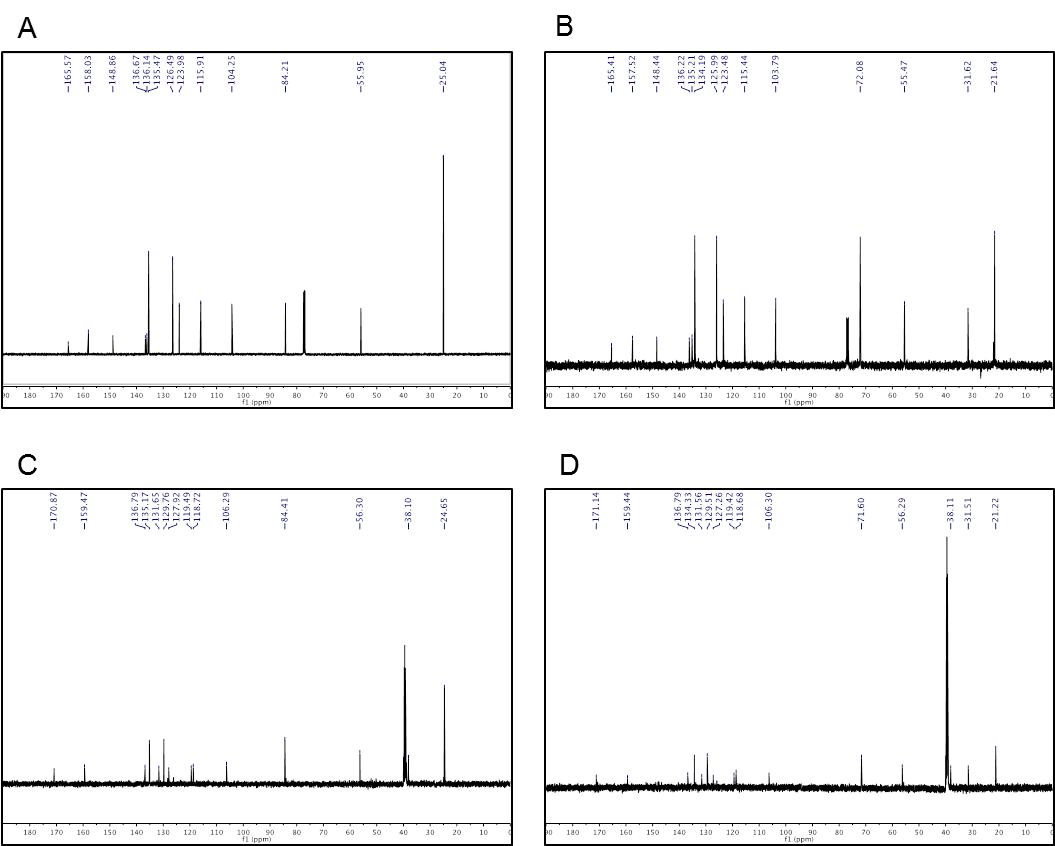


**Supplementary Figure 2.**^13^C NMR spectra of **A)** BE01 **B)** BE02 **C)** mBE01 and **D)** mBE02


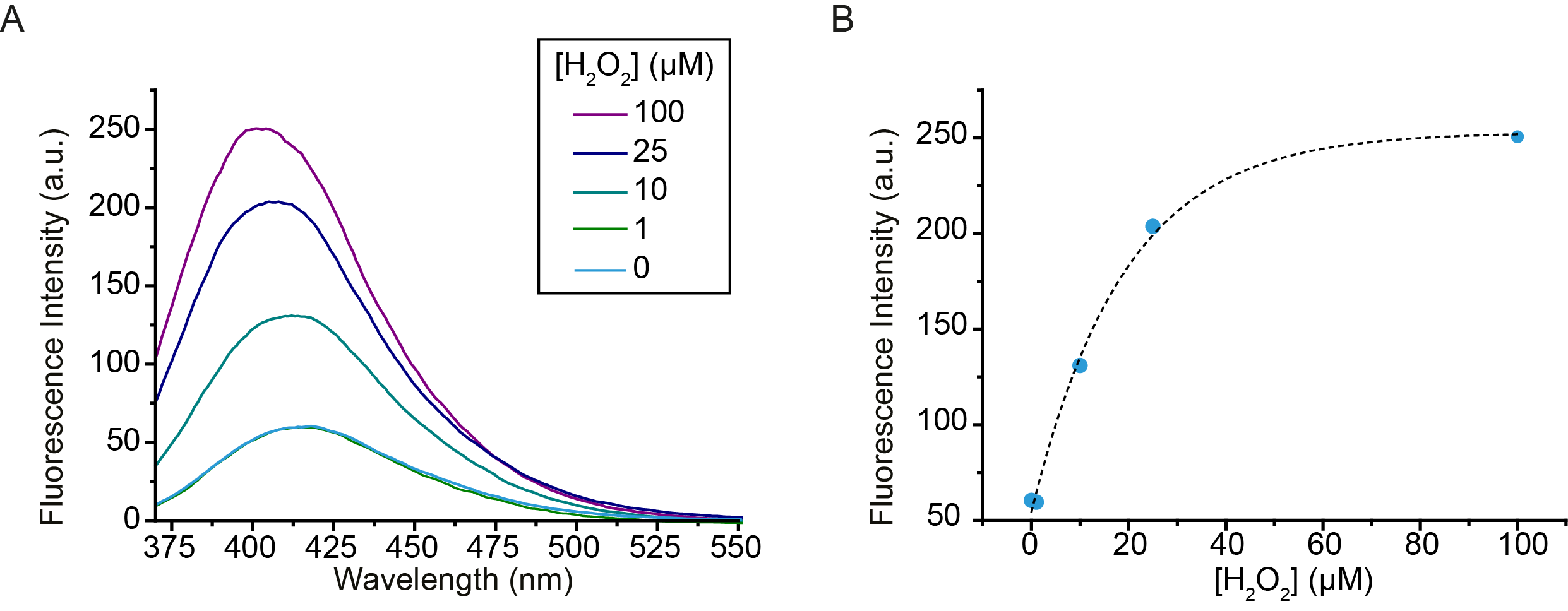


**Supplementary Figure 3. A)** Fluorescence spectra of 5 µM BE01 following 5 h incubation with H_2_O_2_ at concentrations from 0-100 µM. **B)** Fluorescence intensity *vs.* H_2_O_2_ concentration curve of the 5 µM BE01 after 5 h incubation.

|  |  | **Hydrolyzed** | | | **Boronate ester** | | | **Oxidized** | | |
| --- | --- | --- | --- | --- | --- | --- | --- | --- | --- | --- |
|  | **Time point**  **(minutes)** | **Mass (gmol^‑1^)** | **Retention**  **Time (minutes)** | **Rel. Area**  **(%)** | **Mass (gmol^‑1^)** | **Retention**  **Time (minutes)** | **Rel. Area**  **(%)** | **Mass (gmol^‑1^)** | **Retention**  **Time (minutes)** | **Rel. Area**  **(%)** |
| BE01 | 0 | 286.26 | 1.88 | 65 | 368.24 | 3.34 | 35 |  |  |  |
| BE01 | 60 | 286.18 | 1.88 | 47 | 368.40 | 3.34 | 53 |  |  |  |
| BE01 and 100 μM H_2_O_2_ | 60 |  |  |  |  |  |  | 258.14 | 2.04 | 100 |
| BE02 | 0 | 286.26 | 1.88 | 100 |  |  |  |  |  |  |
| BE02 | 60 | 286.26 | 1.88 | 100 |  |  |  |  |  |  |
| BE02 and 100 μM H_2_O_2_ | 60 | 286.34 | 1.88 | 10 |  |  |  | 258.22 | 2.04 | 90 |
| mBE01 | 0 | 300.28 | 0.49 | 100 |  |  |  |  |  |  |
| mBE01 | 60 | 300.28 | 0.49 | 100 |  |  |  |  |  |  |
| mBE01 and 100 μM H_2_O_2_ | 60 | 300.12 | 0.49 | 27 |  |  |  | 272.24 | 0.58 | 64 |
| mBE02 | 0 | 300.20 | 0.49 | 100 |  |  |  |  |  |  |
| mBE02 | 60 | 300.28 | 0.49 | 100 |  |  |  |  |  |  |
| mBE02 and 100 μM H_2_O_2_ | 60 | 300.28 | 0.49 | 12 |  |  |  | 272.32 | 0.58 | 70 |

|  |  | **Oxidized, de-methylated** | | |
| --- | --- | --- | --- | --- |
|  | **Time point**  **(minutes)** | **Mass (gmol‑1)** | **Retention**  **Time (minutes)** | **Rel. Area**  **(%)** |
| mBE01 and 100 μM H_2_O_2_ | 60 | 258.22 | 2.04 | 9.47 |
| mBE02 and 100 μM H_2_O_2_ | 60 | 258.22 | 2.04 | 18.63 |

Mass = mass detected with LCMS; Rel. Area = relative area under the curve in the UV chromatogram (monitored at 254 nm). Hydrolysed = boronic acid derivative of dye; Boronate ester = dye with boronate ester functionality; Oxidized = phenol derivative of the dye after oxidative cleavage. Oxidized, de-methylated = phenol derivative of the dye after oxidative cleavage and loss of methyl group on Nitrogen.

**Supplementary Table 1**. Assessing the reaction of the four dyes with H_2_O_2_ via Liquid chromatography–mass spectrometry (LCMS).

**Confirmation of reaction with H_2_O_2_** **via LCMS**

LCMS analysis of the reaction of the dyes with H_2_O_2_ was performed using a Waters Acquity H-class UPLC coupled with a single quadrupole Waters SQD2. ACQUITY UPLC CSH C18 Column, 130 Å, 1.7 µm, 2.1 mm × 50 mm was used as the UPLC column. The conditions of the UPLC method are as follows:

Solvent A: Water +0.1% Formic acid; Solvent B: Acetonitrile +0.1% Formic acid; Gradient of 0-4 minutes 5% - 100%B + 1 minute 100% B with re-equilibration time of 2 minutes. Flow rate: 0.6 ml/min; Column temperature of 40^o^C; Injection volume of 2 mL. The signal was monitored at 254 nm.

Samples were prepared by diluting the stock solutions of the BE and mBE compounds (prepared as previously described) into PBS (pH 7.4) to a concentration of 30 µM. The diluted dye solutions were analysed via LCMS directly after dilution (t = 0 hour) as well as 1 hour after dilution (t = 1 hour) (Supplementary Table 1). To proof the oxidative cleavage of the boronic esters to a phenol triggered by H_2_O_2_, the diluted dye solutions were exposed to 100 µM H_2_O_2_ and analysed via LCMS after 1 hour of incubation (Supplementary Table 1).

The LCMS data confirm that after 1 hour of incubation with 100 µM H_2_O_2_ the majority of the dye molecules were converted into the phenol (Supplementary Table 1 BE01 100%; BE02 90%; mBE01: 73%; mBE02 88%). As opposed to this no phenol formation was detected after 1 hour without H_2_O_2_ reinforcing that the changes observed in the optical measurements are based on the oxidative cleavage of the boronic esters by H_2_O_2_.

The detection of the hydrolysed species of the dyes by LCMS at t = 0 as well as after 1 hour without H_2_O_2_ is likely due to on-column hydrolysis of the boronic esters, which is promoted by the acidic pH of the mobile phase, the column as well as the column temperature (1), as well as partial in situ hydrolysis of the esters under the aqueous conditions (2). However, the influence of the partial in situ hydrolysis on the sensing of H_2_O_2_ can be neglect since it does not impact the fluorescence based measurements (no significant change in fluorescence over 300 minutes in PBS; results shown in Supplementary Figure 5). LCMS analysis of the samples diluted in MeCN instead of PBS did also lead to the detection of the hydrolysed species of the dyes, confirming the contribution of on-column hydrolysis. Further LCMS and NMR studies in DMSO as well as D_2_O/d-DMSO reinforcing the stability of the four dyes under these conditions.


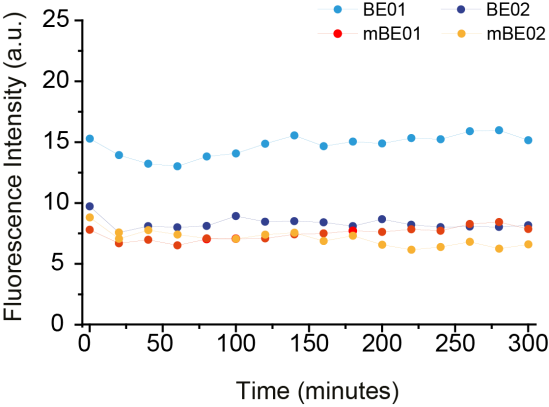


**Supplementary Figure 4.** Fluorescence *vs.* time kinetics of the BE and mBE dyes (5 μM).


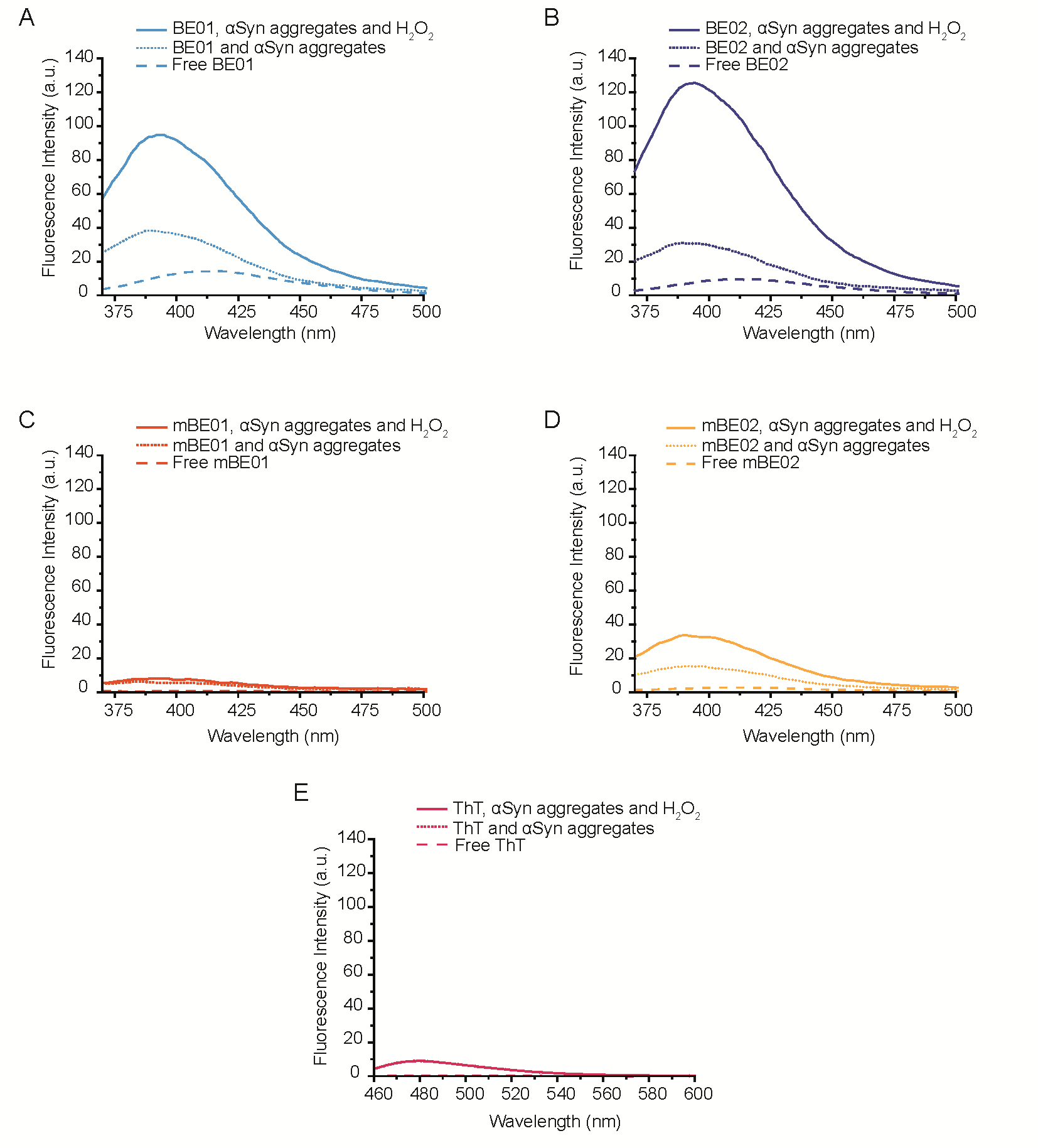


**Supplementary Figure 5.** Bulk fluorescence spectra of **A)** BE01, **B)** BE02, **C)** mBE01, **D)** mBE02 and **E)** ThT free in PBS buffer, with αSyn aggregates and with concomitant αSyn aggregates and H_2_O_2_.

|  | **BE01** | **BE02** | **mBE01** | **mBE02** | **ThT** |
| --- | --- | --- | --- | --- | --- |
| **Free dye/H2O2** | 0.01570 | 0.3320 | 0.0120 | 0.0020 | 0.5000 |
| **Free dye/ αSyn** | 0.00110 | 0.0030 | 0.0070 | 0.0160 | 0.0001 |
| **Free dye/H2O2 and αSyn** | 0.00003 | 0.0001 | 0.0007 | 0.0003 | 0.0001 |

**Supplementary Table 2.** Two tailed p-value results from statistical analysis of bulk fluorescence characterisation data (Figure 2D).

**
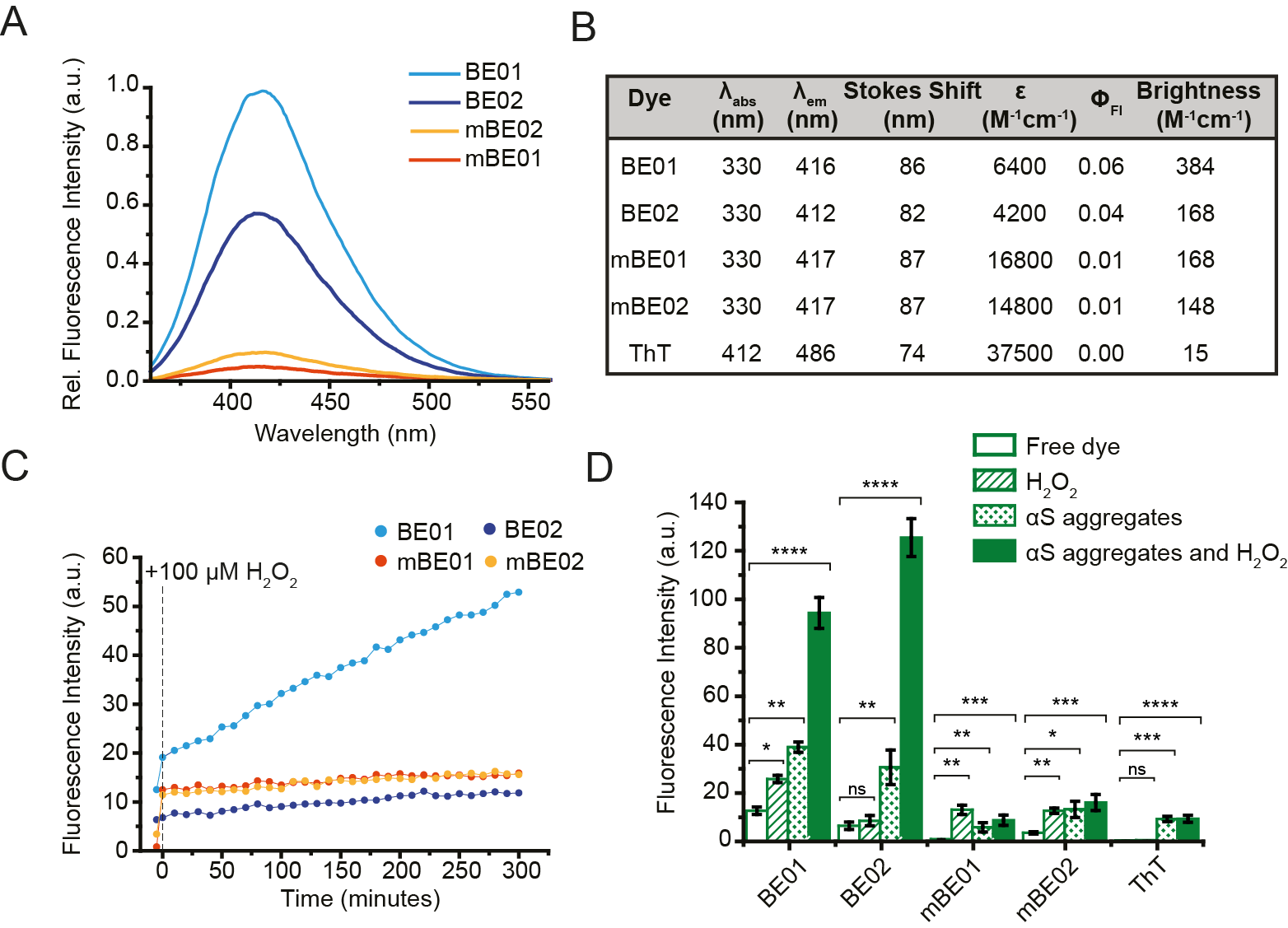
**

**Supplementary Figure 6.** Bar graph of photophysical properties (Figure 2D) with illustration of levels of significance following statistical testing.


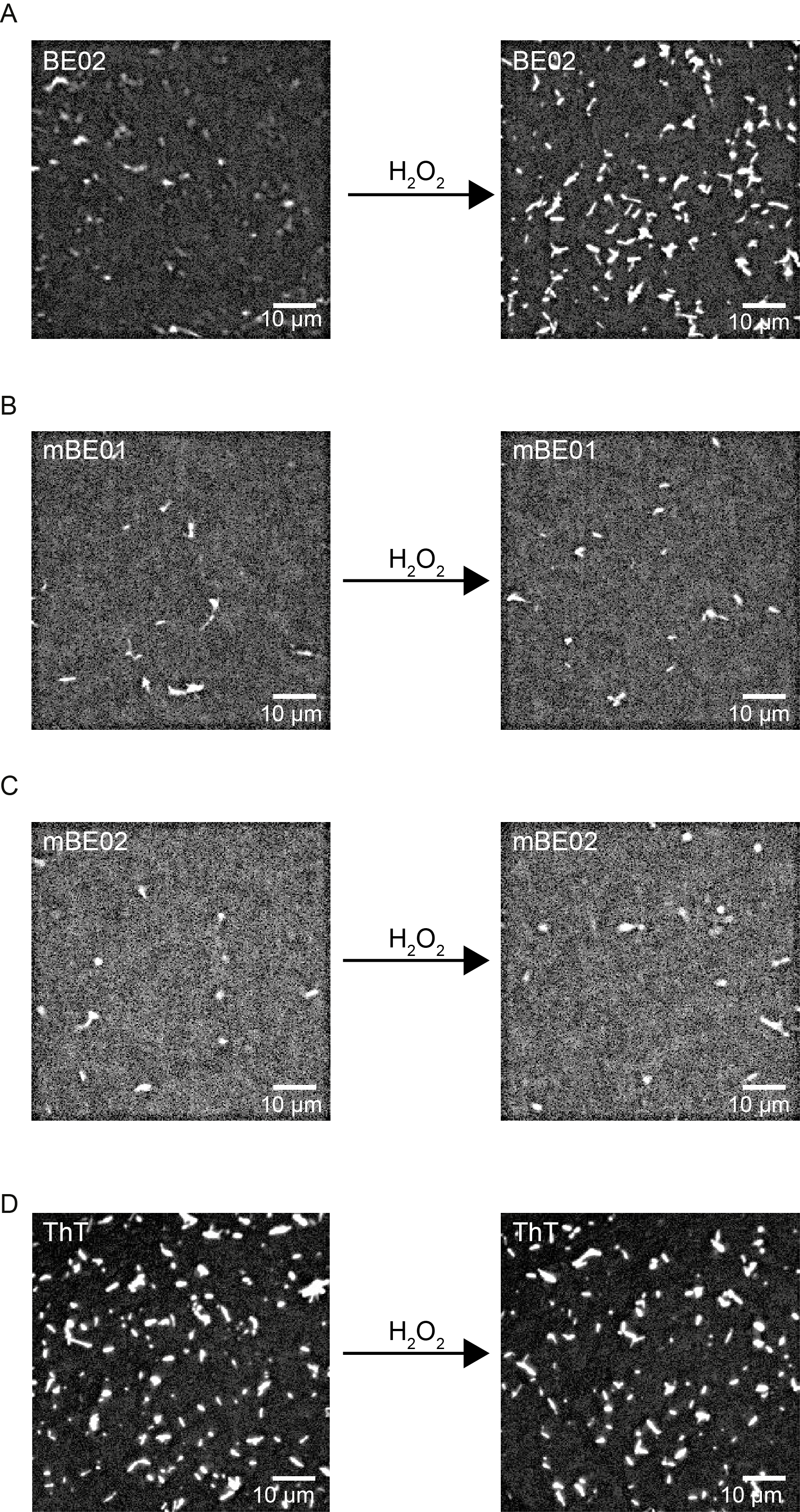


**Supplementary Figure 7.** Representative background subtracted average intensity SAVE images of **A)** BE02, **B)** mBE01, **C)** mBE02.


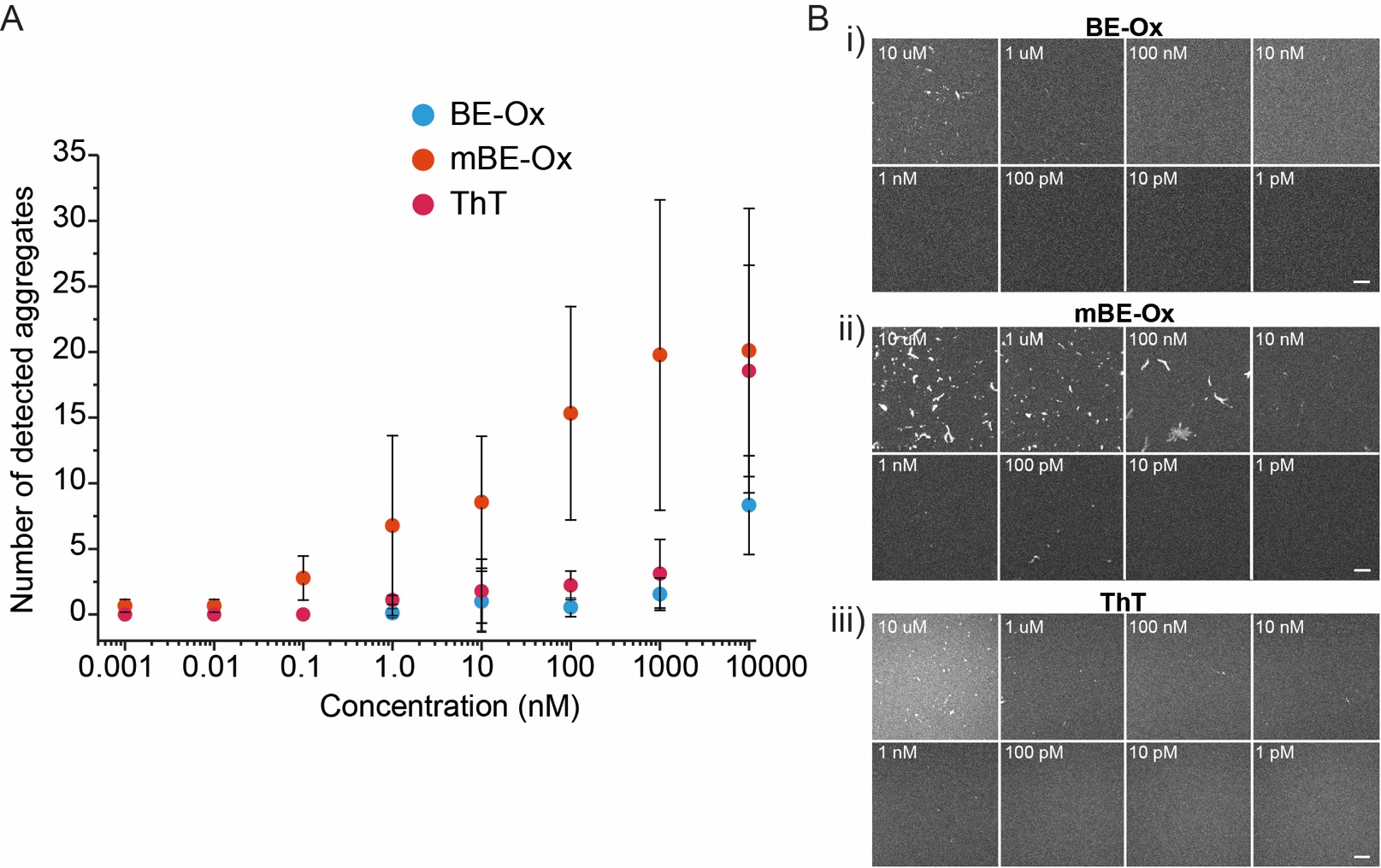


**Supplementary Figure 8.** Determination of the limit of detection of oxidised BE and mBE bifunctional probes and ThT **A)** A plot of the number of single αSyn aggregates detected per field of view against concentration of dye. **B)** Representative SAVE images of immobilized αSyn aggregates with 10 μM-1 pM i) BE-Ox, ii) mBE-Ox, iii) ThT Scale bar = 10 µm.


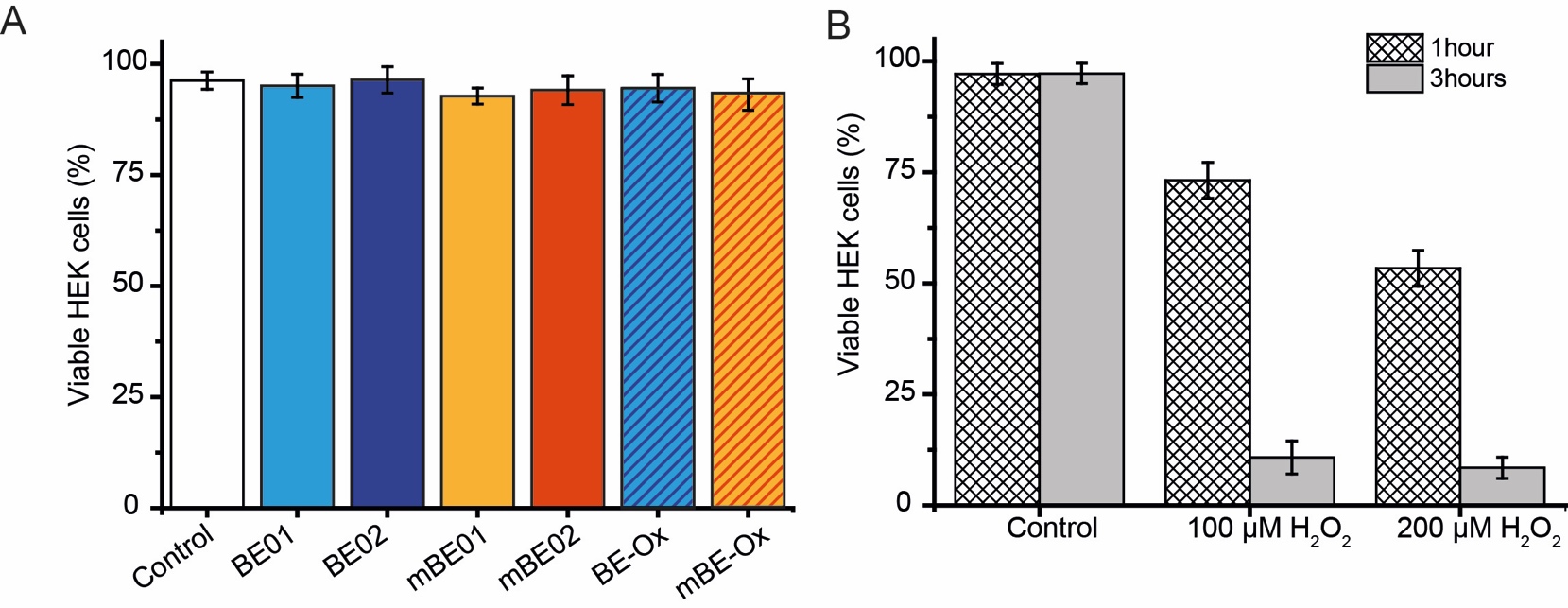


**Supplementary Figure 9. A)** . a) Bar graph illustrating percentage of viable HEK293 cells after a 24 hour treatment with BE and mBE probes b) HEK293 viability after 1 hour and 3 hour treatment with H_2_O_2_.
